# Medical negligence: Criminal prosecution of medical professionals, importance of medical evidence: Some guidelines for medical practitioners

**DOI:** 10.4103/0970-1591.56207

**Published:** 2009

**Authors:** M. S. Pandit, Shobha Pandit

**Affiliations:** Advocates & Corporate Legal Adviser, D – 29, 5^th^ Floor, Mantri Kishor Park, Bhosale Nagar, Pune 411 007, India

**Keywords:** Criminal negligence, medical negligence, medical literature

## Abstract

The changing doctor-patient relationship and commercialization of modern medical practice has affected the practice of medicine. On the one hand, there can be unfavorable results of treatment and on the other hand the patient suspects negligence as a cause of their suffering. There is an increasing trend of medical litigation by unsatisfied patients. The Supreme Court has laid down guidelines for the criminal prosecution of a doctor. This has decreased the unnecessary harassment of doctors. As the medical profession has been brought under the provisions of the Consumer Protection Act, 1986, the patients have an easy method of litigation. There should be legal awareness among the doctors that will help them in the proper recording of medical management details. This will help them in defending their case during any allegation of medical negligence.

## INTRODUCTION

Doctors and patients are not generally seen as adversaries. Actually, doctors are generally seen as healers and saviors. At the same time, the livelihood of doctors and the medical fraternity depends on patients. In other words, doctors are there because patients need them. But with commercialization, this relationship has not retained the age old sanctity that is a matter of great concern to the medical profession. Medical professionals were greatly agitated when it was held that the services rendered by the medical fraternity are covered under the Consumer Protection Act. One of the reasons was undoubtedly a small percentage of failures of treatment that dogs the profession despite due care and caution. Just as rain drops result in a stream, a small percentage of unsuccessful cases may result in good number of dissatisfied patients and a plethora of cases against a hospital or a doctor taking a toll of its or his time and hard earned reputation. Secondly, the ease with which a consumer case can be filed is likely to encourage frivolous and speculative complaints intended to exploit the consumer jurisdiction. The apex court has recognized this fact and ruled against criminal prosecution of doctors unless gross negligence is established. The following are some of the issues of medical negligence along with some landmark decisions.

## CRIMINAL PROSECUTION OF DOCTORS

Doctors can be prosecuted for obvious criminal activity like violations of statutory provisions of Acts like the Transplantation of Human Organs Act. The newspapers tell us that the first conviction for fetal sex determination has sent shock waves throughout the country. According to these reports, a sub divisional judicial magistrate in Haryana sentenced a doctor and his assistant to 2 years imprisonment and fine of Rs. 5000/- each for violating the Pre-Natal Diagnostic Techniques (Regulation and Prevention of Misuse) Act, 1994. It is expected that weeding out the black sheep in the profession will go a great way in restoring the honor and prestige of a large number of doctors and hospitals who are devoted to the profession and scrupulously follow the ethics and principles of this noble profession.

However, it is common to implicate doctors in criminal cases alleging negligence in the death of a patient under treatment. In the case of Dr. Suresh Gupta's case (Dr. Suresh Gupta *vs*. Govt of NCT Delhi, AIR 2004, SC 4091: (2004)6 SCC 42), the Full bench of the Supreme Court of India consisting of Chief Justice R.C. Lahoti, Justice G.P. Mathur, and Justice P.K. Balasubramanyam declared while reviewing the previous order that extreme care and caution should be exercised while initiating criminal proceedings against medical practitioners for alleged medical negligence. In a well considered order, the apex court felt that bonafide medical practitioners should not be put through unnecessary harassment. The court said that doctors would not be able to save lives if they were to tremble with the fear of facing criminal prosecution. In such a case, a medical professional may leave a terminally ill patient to his own fate in an emergency where the chance of success may be 10% rather then taking the risk of making a last ditch effort towards saving the subject and facing criminal prosecution if the effort fails. Such timidity forced upon a doctor would be a disservice to society. The court held that simple lack of care, error of judgment, or an accident is not proof of negligence on the part of a medical professional and that failure to use special or extraordinary precautions that might have prevented a particular incidence can not be the standard for judging alleged medical negligence.

The apex court laid down the following guidelines regarding prosecution of cases: cases of doctors being subjected to criminal prosecution are on the increase. The criminal process once initiated subjects in the medical professionals to serious embarrassment and sometimes harassment. Statutory rules or executive instructions incorporating certain guidelines are to be framed and issued by the Government or State Government in consultation with the Medical Council of India. A private complaint may not be entertained unless the complainant produces prima facie evidence before the court in the form of credible opinion given by another competent doctor to support the charge of rashness or negligence on the part of the accused doctor.

In the case of Jacob Mathew (Dr.) *vs*. State of Punjab and Anr. III (2005) CPJ 9 (SC) (Criminal Appeal) where a cancer patient in an advanced stage died due to non availability of an oxygen cylinder in the room, the Supreme Court considered three weighty issues: first, negligence in the context of the medical profession necessarily calls for treatment with difference; second, the difference between occupational negligence and medical negligence has to be properly understood; and third the standard to be applied to hold a medical professorial as negligent has to be carefully considered. The apex court further held that there is no case that the accused doctor was not a qualified doctor to treat the patient was made and therefore the accused appellant can not be prosecuted under Section 304 A of IPC for the non availability of an oxygen cylinder though he may be liable under civil law.

## IN A HIGH RISK CASE, ACCIDENTAL EVENTUALITY CANNOT ALWAYS BE CONTROLLED, HENCE CONCLUSION OF DEFICIENCY IN SERVICE CANNOT BE DRAWN

In the case of Mrs. Shantaben Muljibhai Patel and Ors. *vs*. Breach Candy Hospital and Research Centre and Ors. I(2005) CPJ 10 (NC), the National Commission dismissed the complaint filed by the wife and 2 sons of the deceased Mr. M.M. Patel who died in Breach Candy Hospital alleging negligence by staff, treatment administered by Dr. Bhattacharya, surgeon, and Dr. Mahatre, Anaesthetist. The claim was for an exorbitant amount of Rs. 1.02 Crores towards the death of the patient, Rs. 2.50 lacs towards hospitalization charges, Rs. 25,000/- towards funeral expenses, Rs. 90 lacs on account of loss due to death arising out of business, and Rs. 25,000/- towards legal expenses. The deceased had undergone a by-pass surgery in 1988 when the mitral valve was replaced. Deterioration of 15% ejection fraction (pumping efficiency) was found in 1996. An operation was performed successfully. While undertaking post-operative treatment, there was extubation of the endotracheal tube. As per medical literature, such chances of extubation are between 8.5% to 13%. Extubation was swift and sudden and was immediately noticed by the duty nurse. An expert doctor was called but intubation was found difficult and the patient died due to cardiac arrest. The hospital was equipped with the necessary equipment and doctors performed their duties to the best of their ability with due care and caution. The deceased was a high-risk case and such accidental eventuality cannot be controlled. Every surgical operation is attended by risk. If something goes wrong, conclusion of deficiency in service can not be drawn. Reasonable care was exercised by the nursing staff. There was no negligence or deficiency in the service provided. While delivering its judgment, the National Commission has approvingly referred to the observations of Lord Denning in Roe and *Woolley vs*. The Ministry of Health and An Anaesthetist, (1954) 2 All ER 131: “Every surgical operation is attended by risks. We can not take the benefits without taking the risks. Every advance in technique is also attended by risks. Doctors like the rest of us, have to learn by experience; and experience often teaches in a hard way.”

A reference was also made to observations of Justice Mc Nair in Bolam *vs*. Friern Hospital Management Committee (1957) 2 All ER 118 wherein a patient suffering from mental illness was advised to undergo electro –convulsive therapy though the therapy has risk of fracture: “In the case of a medical man negligence means failure to act in accordance with the standards of reasonably competent medical men at the time. This is a perfectly accurate statement, as long as it is remembered that there may be one or more perfectly proper standard; and if a medical man confirms to one of those proper standards then he is not negligent. Counsel for the plaintiff was also right in my judgment in saying that mere personal belief that a particular technique is best is no defense, unless that belief is based on reasonable grounds. That again is unexceptionable.” A doctor is not guilty of negligence if he has acted in accordance with a practice accepted as proper by a reasonable body of medical men skilled in that particular art.”

The National Commission in S.R. Shivaprakash and Ors. *vs*. Wochardt Hospital Ltd. and Ors, II(2006)CPJ123(NC) wherein a patient died due to sudden extubation of an endotracheal tube due to sudden violent coughing resulting in cardiac arrest 2 days after surgery held that the operation involved extremely high risk, all necessary care taken for saving patient's life, such displacement is nothing new to medical profession. The main cause for the patient's death is the poor pre-operative inherent condition of the patient, negligence of doctors/nurses not made out, principle of res ipsa loquitor as alleged not inferred, hence opposite parties (hospital and doctors) are not liable. In so far as non supply of relevant documents it was held that doctors and hospital are obliged to supply all records containing treatment given, medicines administered, and nature of operation.

## IN A CASE WHERE MEDICAL RECORDS AND CONSENT OBTAINED FROM A PATIENT WERE NOT PRODUCED, MEDICAL NEGLIGENCE WAS ESTABLISHED BASED ON THE PRINCIPLE OF *RES IPSA LOQUITUR*

In the case of Dr. Shyam Kumar *vs*. Rameshbhai Harmanbhai Kachhiya, I(2006)CPJ16(NC) the National Commission held that an operation was conducted for glaucoma and cataract but the retina was weakened and eye sight was lost, it was held that conducting the operation without obtaining informed consent was improper. A patient can not be deprived of this information. Not even medical records were produced, hence the patient is entitled to claim compensation.

## MEDICAL LITERATURE

In the case of P.Venkata Laskhmi *vs*. Dr. Y. Savita Devi, II(2004)CPJ14(NC), the National Commission found merit in the contention of the appellant that medical literature filed by Complainant was not considered. The brief facts of the case were: ‘complications developed at delivery time – the baby had breathing problems and other related problems and was shifted to another hospital. Negligence was alleged against the attending doctors – the complaint was dismissed by the State Commission on the ground that negligence was not proved by expert evidence, hence an appeal. Medical literature produced by the complainant was not considered by the State Commission. The matter was remanded back for re-hearing.

The National Commission inter alia observed as follows: “Absence of expert evidence has also been referred to as one of the grounds of ‘no-proof’ of negligence. We have seen the material on record and find that while no expert evidence has been produced or examined by the appellant, we have to see this in ‘ground reality’ terms, that very rarely, if ever, any other doctor comes forward to give evidence in person or by way of evidence against other doctor. In this case, this gap was made good by producing literature on all the points at issue.”

## HOSPITAL RECORDS

In the case of Meenakshi Mission Hospital and Research Centre *vs*. Samuraj and Anr., I(2005) CPJ 33 (NC), the National Commission held the hospital guilty of negligence on the grounds that the name of the anesthetist was not mentioned in the operation notes though anesthesia was administered by two anesthetists at 10 a.m and 10.30 a.m. The child was pulseless and the doctor who administered anesthesia was not produced before the Commission. Two progress cards about the same patient on two separate papers were produced. What the two anesthetists were doing inside the O.T was not explained. The hospital is accountable for whatever happens in the hospital and was held liable to pay the compensation and cost. It is relevant to note that in this case the District Forum found the hospital negligent and awarded a compensation of Rs. 3 lacs and cost of Rs. 2000/-. Thereafter, the State Commission had dismissed the appeal with a cost of Rs. 500.

## CIRCUMSTANCES

Transparency in dealings will always help in litigation. As the medical fraternity is not used to detailed record keeping, doubts do arise in the course of legal proceedings. Hospitals and doctors may be held negligent or otherwise based on the facts and circumstances even in the absence of conclusive evidence. Thus, in the case of Dr. Sham Lal and Ors. *vs*. Mrs. Saroj Rani and Ors. 1 (2003) CPJ 47 (NC), the National Commission considered the question whether an intra muscular injection administered intravenously caused an embolism of the heart resulting in the death of the patient.

Cross Appeals were filed by both the parties in this matter and the dispute was adjudicated based on expert evidence and circumstantial evidence. The deceased Mr. Ravinder Kumar and his wife went to Dr. Soni, their physician for a check-up and treatment. The late Mr. Ravinder Kumar complained to the Doctor (Dr. Soni) about pain in the abdomen which started the night before. The doctor diagnosed this to be a case of left renal colic and prescribed the following medicines: Cap. Spasm Poxvan (Pain killer) Inj. Prolution, Lig. Citrika, and Calcurosin. The doctor advised that the injection be taken intramuscularly. The injection was purchased from the chemist and it was advised to get it injected from the chemist's father, Dr. Shem Lal who runs a clinic adjacent to the chemist shop. This injection was administered intravenously as a result of which the body started trembling and within a few minutes Ravinder Kumar died on the spot. The State Commission, after hearing the parties, awarded a compensation of Rs.6 lakhs; Rs.2 lakhs to the complainant, widow of the late Shri Ravinder Kumar, Rs.1 lakh each to 3 children, and Rs.50,000/- each to the parents and the cost of Rs.5000/-.

The National Commission made the following observations: 1) as per the expert opinion again un-rebutted that if the injection Prolusion Depot is administered intravenously, it stops blood supply to the heart, 2) Dr. Soni in his affidavit has stated that he prescribed these medicines, after which the deceased never came back. Dr. Soni has not been examined at all, 3) there are affidavits of Shri Jagir Singh and Jagannath stating that they saw the injection being administered on the deceased person, 4) on the question of whether “Prolusion Depot” was at all injected, the post mortem report is clear that there was a 1.5 cm circular reddish mark on the deceased, and 5) it was denied by the 1^st^ Respondent (Dr. Sham Lal) in his affidavit that any injection at all was administered to the deceased.

In the absence of direct evidence, the first and third Respondents were held negligent based on facts and circumstances. The National Commission found no merit in the appeal excepting that no negligence was found against Resp No. 2. Even the appeal filed for enhancement of compensation was rejected except that the complaints were awarded with interest at 12% p.a. on the amount of compensation from the date of filing the complaint. Thus, the appeal was allowed in part and the order of the State Commission was modified to that extent.

## EXAMINATION OF WITNESSES IN A MEDICAL NEGLIGENCE CASE

The Consumer Forum has to be considerate while dealing with a request of a complainant to examine the doctor or a medical professional. The Forum may devise a convenient method like appointment of commissioner or direct the applicant to put the questions by way of interrogatories and the concerned doctor to file his reply by way of an affidavit.

In the case of Dr. J.J.Merchant and Ors. *vs*. Shreenath Chaturvedi, 2002 (4) ALL M.R 605 (S.C), the Supreme Court held that merely because it is mentioned that the Commission or the Forum is required to have a summary trial, it would hardly be grounds for directing the consumer to approach the Civil Court. It would also be completely wrong to assume that because a summary trial is provided, justice cannot be done when some questions of facts are required to be dealt with or decided. The Act provides sufficient safeguards.

Further, as observed by the apex court, S.13(4) of the Act empowers the Commission to exercise the powers vested in the Civil Court for discovery and production of any document, the reception of evidence on affidavit and of issuing of any commission qua examination of any witness. The Commission can always insist on production of all documents relied upon by the parties along with the complaint and the defense version. Furthermore, as observed by the apex court, the Commission has discretion to examine the experts if required in an appropriate matter. In cases where it is deemed fit to examine experts, recording of evidence before a Commission may be time-consuming. The Act specifically empowers the Consumer Forums to follow the procedure that may not require more time or delay the proceedings. Caution is required to strictly follow the procedure.

Under the Act, while trying a complaint, evidence could be taken on affidavits [under section 13(4)(iii)]. It also empowers such Forums to issue any commission for examination of any witness [under section 13(4)(v)]. It is also to be stated that Rule 4 in Order XVIII of C.P.C. is substituted which inter alia provides that in every case, the examination-in-chief of a witness shall be on affidavit and copies thereof shall be supplied to the opposite party by the party who calls him for evidence. It also provides that witnesses could be examined by the Court or the commissioner appointed by it. The Commission is also empowered to follow the procedure. The affidavits of the experts including the doctors can be taken as evidence. Thereafter, if cross-examination is sought for by the other side and the Commission finds it proper, it can easily evolve a procedure permitting the party who intends to cross-examine by putting certain questions in writing and those questions also could be replied by such experts including doctors on affidavits. In a case where stakes are very high and the party intends to cross-examine such doctors or experts, there can be video conferences or questions asked by arranging a telephone conference and at the initial stage this cost should be borne by the person who claims the video conference. Furthermore, cross-examination can be undertaken by the Commissioner appointed at the working place of such experts at a fixed time. Thus, as held by the Supreme Court in Dr. J.J. Merchant and Ors., an interrogatory or questionnaire may be prepared by the Applicant in lieu of a cross examination of a witness who is not in a position to be present.

## DEFENSE OF MEDICO-LEGAL CASES BY DOCTORS

When a legal notice is received or a consumer case alleging deficiency in service is filed against a doctor, it creates a lot of emotional disturbance as the reputation of medical professionals is built over years through sheer hard work, expertise, and skill acquired by strenuous training and investment over the years. Nevertheless, hospitals and doctors have to keep in mind that as per the decision of Supreme Court in a catena of cases, the medical profession is finally held to be covered and that there is no easy escape on technical grounds. They have to take care of the legal requirements and face the situation head-on. One should not forget that it is very important to reply to a legal notice in a very thorough manner. A well prepared reply will serve as the basis of a Written Statement to be filed in case a Consumer Complaint is instituted against the doctor and/or hospital. Quite often a well prepared notice reply drives home the fact that the hospital and doctor will not succumb to pressure and speculative cases will be fought hard. Our personal experience is that in a good number of cases a well prepared notice reply achieves the desired result. In case an unjustified, false, or speculative consumer case is filed alleging deficiency in service rendered by a hospital or a doctor, they have to take care of the requirements of the law such as the timely filing of a written statement, affidavit etc., and put up a good defense at the time of the hearing. Most importantly, case history, indoor case papers, clinical records, report of investigation if any, affidavit of dealing doctors, X-rays, test results etc., will be of immense help.

Special attention has to be given to expert evidence of a qualified and independent medical professional. It is most important to keep in mind that the complainant has to establish that the manner and nature of treatment (including pre- and post-operative care) was so deficient that it falls short of the skill and /or standard expected from average medical practitioners and not that of a highly qualified and exceptionally gifted person. While coming to such a conclusion, a Consumer Forum has to keep in mind the qualification, infrastructure, and facilities available at such a place. The defending doctor or hospital has to apprise the Consumer Forum about the accepted practice in treatment, negligence on the part of patient in availing treatment promptly, or following medical advice etc. Needless to mention medical professionals need to maintain a good relationship with their brothers in the profession. Attention has to be given to corroborative medical literature on the subject. Lastly, relevant case law on the subject will also be helpful. In case a hospital or a doctor finds itself or himself in a situation where it is very likely such an act falls under the category of medical negligence, say res *ipsa loquitur* or *negligent per se*, e.g., a case in which a wrong limb or organ was treated, operated, amputated, or infected blood was given or qualification was wrongly written etc., such a case does not need any special evidence to establish and it is advisable that such a claim is compromised after taking the insurer into confidence. Furthermore, the doctor and/or hospital are entitled to engage the services of a lawyer to represent them in the matter.

There is no denying the fact that prolonged litigation adversely affects the reputation of a doctor or hospital even though he/it eventually wins the case. The medical fraternity is concerned with safeguards against speculative and vexatious claims. While one can not deny the fact that there are genuine cases involving medical negligence, the issue that bothers the medical fraternity is that quite often irreparable damage is caused to a doctor or a hospital on account of a large number of speculative complaints.

To summarize, a good defense that has to be put up by the doctor includes the following:

1. Avail the services of a good lawyer. The doctor and /or hospital are entitled to engage the services of a lawyer to represent them in the matter.
2. Timely filing of a written statement, affidavit, and all other documents as required.
3. It is important to properly maintain case history, clinical records, affidavit of all the treating doctors, X-rays, laboratory test results, etc. which will be of immense help in supporting the doctor's claim.
4. Special attention has to be given to bring in the expert evidence of a qualified and independent medical professional. It is advisable to file an affidavit of the expert as well.
5. Corroborative medical literature on the subject should be submitted.
6. Relevant case law on the subject will also be helpful.
7. Put up a good defense at the time of the hearing.

